# Effect Size Guidelines, Sample Size Calculations, and Statistical Power in Gerontology

**DOI:** 10.1093/geroni/igz036

**Published:** 2019-09-04

**Authors:** Christopher R Brydges

**Affiliations:** Department of Human Development and Family Studies, Colorado State University, Fort Collins

**Keywords:** Effect size, Sample size, Statistical power, Statistical significance

## Abstract

**Background and Objectives:**

Researchers typically use Cohen’s guidelines of Pearson’s *r* = .10, .30, and .50, and Cohen’s *d* = 0.20, 0.50, and 0.80 to interpret observed effect sizes as small, medium, or large, respectively. However, these guidelines were not based on quantitative estimates and are only recommended if field-specific estimates are unknown. This study investigated the distribution of effect sizes in both individual differences research and group differences research in gerontology to provide estimates of effect sizes in the field.

**Research Design and Methods:**

Effect sizes (Pearson’s *r*, Cohen’s *d*, and Hedges’ *g*) were extracted from meta-analyses published in 10 top-ranked gerontology journals. The 25th, 50th, and 75th percentile ranks were calculated for Pearson’s *r* (individual differences) and Cohen’s *d* or Hedges’ *g* (group differences) values as indicators of small, medium, and large effects. A priori power analyses were conducted for sample size calculations given the observed effect size estimates.

**Results:**

Effect sizes of Pearson’s *r* = .12, .20, and .32 for individual differences research and Hedges’ *g* = 0.16, 0.38, and 0.76 for group differences research were interpreted as small, medium, and large effects in gerontology.

**Discussion and Implications:**

Cohen’s guidelines appear to overestimate effect sizes in gerontology. Researchers are encouraged to use Pearson’s *r* = .10, .20, and .30, and Cohen’s *d* or Hedges’ *g* = 0.15, 0.40, and 0.75 to interpret small, medium, and large effects in gerontology, and recruit larger samples.

**Translational Significance**

This study examines statistical power (the probability of observing a true effect) and finds that research in the field of Gerontology reveals small effect sizes leading to some studies being underpowered to detect true effects. By increasing statistical power in accordance with expectable effect sizes, researchers can be confident that true effects are detectable and findings are replicable across studies.

It is recommended that researchers report effect sizes (Wilkinson & the Task Force on Statistical Inference, 1999) as they can provide valuable additional information regarding a test result that traditional null hypothesis significance testing cannot, such as the magnitude of a difference or association. These statistics are commonly presented as a standardized mean difference (ie, Cohen’s *d* or Hedges’ *g*) or as the strength of association (Pearson’s *r*) between two groups or variables. Cohen (1988, 1992) provided guidelines for the interpretation of these values: values of 0.20, 0.50, and 0.80 for Cohen’s *d* and Hedges’ *g* are commonly considered to be indicative of small, medium, and large effects (.10, .30, and .50, respectively, for Pearson’s *r*). However, these interpretations were not based on formal statistical analyses of data, and it is feasible that the distribution of effect sizes could vary between fields of research (Hemphill, 2003). In fact, Cohen (1988, 1992) suggested that a medium effect size should be observable to the naked eye, which may be unrealistic given the range of research areas that use the aforementioned guidelines. Furthermore, Cohen (1988, 1992) stated that these guidelines should only be used if estimates specific to the research area of interest are unknown.

Research examining effect size distributions in various fields of research have found considerable variability from these estimates, with small, medium, and large effect sizes defined as the 25th, 50th, and 75th percentiles of all effect size values in each case, respectively. Gignac and Szodorai (2016) reported small, medium, and large correlations of .11, .19, and .29 in individual differences research in psychology, and Quintana (2017) observed Cohen’s *d* values of 0.26, 0.51, and 0.88 for small, medium, and large effects in case–control studies of heart rate variability. In addition, Lovakov and Agadullina (2017) reported Hedges’ *g* values of 0.15, 0.38, and 0.69, and Pearson’s *r* values of .12, .25, and .42 in social psychology.

Although effect size distribution is dependent upon outcome measure and population of interest, the variability of the distribution of effect sizes between fields suggests that Cohen’s (1988, 1992) guidelines are potentially inappropriate, which is likely to lead to inaccurate results from a priori power analyses. Statistical power refers to the probability that a test will reject the null hypothesis (ie, report a statistically significant result), assuming there is a true effect of a given size, and it varies as a function of effect size, sample size, and alpha level (typically .05). Power is conventionally set at .80 (Cohen, 1988), which implies that a study investigating a true effect will correctly reject the null hypothesis 80% of the time and will report a false negative (commit a Type II error) in the remaining 20% of cases.

A major issue when designing an informative experiment is choosing a sample size that will ensure sufficient statistical power. Sample size selection depends on several factors (eg, within-subjects vs. between-subjects study design), but sample size should ideally be chosen such that the test has enough power to detect effect sizes of interest to the researcher (Morey & Lakens, 2016). From this, a planned study can potentially be underpowered if the study design is insensitive to the true effect size (ie, if a researcher conducts an a priori power analysis where he/she unknowingly has an incorrect estimation of the effect size of interest). For example, if a social psychologist expects a medium effect size in a study examining differences between two groups (ie, using an independent samples *t*-test), the required sample size to achieve power of .80 with alpha of .05 is *n* = 64 per group when using Cohen’s (1988) estimate of Cohen’s *d* = 0.50. When using Lovakov and Agadullina’s (2017) estimate of Hedge’s *g* = 0.38, however, 110 participants per group are required to achieve power of .80. Assuming the lower estimate is correct, a researcher would only achieve power of .57 if he/she recruited 64 participants per group, per calculations based on Cohen’s estimate. That is, there would only be a 57% probability of correctly rejecting the null hypothesis, which, in turn, may affect the chances of the research being published (Ferguson & Heene, 2012). In addition, underpowered studies are more likely to report an overly inflated effect size (Ioannidis, 2008) through questionable research practices such as *p*-hacking (Simmons, Nelson, & Simonsohn, 2011), which can result in a greater likelihood of failed attempts to replicate the finding (Maxwell, 2004).

Although Cohen’s (1988, 1992) guidelines of effect size distributions are used extensively within behavioral sciences, it is possible that they may not be entirely appropriate for gerontology research. Power analyses and effect size interpretations should be based on empirically observed research. Although research in other fields has reported some deviance from the aforementioned provided estimates (Gignac & Szodorai, 2016; Lovakov & Agadullina, 2017; Quintana, 2017), these data have not been systematically analyzed in gerontology (with the exception of Levenson (1980), who analyzed statistical power in attitude research). By calculating empirically derived effect size distributions, gerontological researchers can design well-powered studies (Isaacowitz, 2018; Pruchno et al., 2015) and gain greater knowledge of their study effects that is guided by previous research in the field.

## Method

The analyses closely followed those of Quintana (2017) and Lovakov and Agadullina (2017). Data, a codebook, R code, a full list of the included meta-analyses, and a preprint of this study are publicly available on the Open Science Framework (https://osf.io/ez367/).

### Search Procedure

Any article with “meta” in the title published in the journals *Journals of Gerontology: Series A, Biological Sciences and Medical Sciences*, *Journal of the American Geriatrics Society*, *The Gerontologist*, *American Journal of Geriatric Psychiatry*, *Journals of Gerontology: Series B, Psychological Sciences and Social Sciences*, *International Journal of Geriatric Psychiatry*, *BMC Geriatrics*, *Aging & Mental Health*, *Geriatrics & Gerontology International*, and *Psychology and Aging* was initially extracted (*n* = 379, as of 2nd May 2019). These 10 journals were chosen as they are the 10 highest-ranked Gerontology journals on Clarivate Analytics’ journal citation ranking for 2017.

### Inclusion and Exclusion Criteria

**Figure 1.**
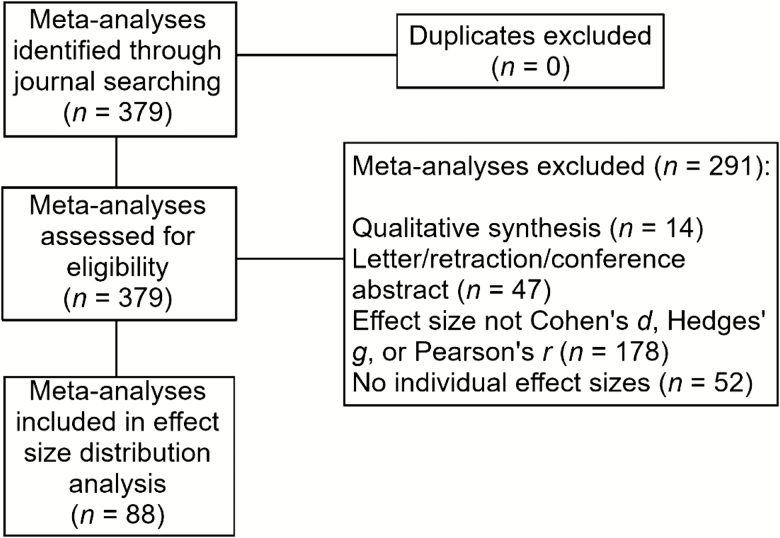
Meta-analysis inclusion flow chart for effect size distribution analysis.

Meta-analyses were included if the results reported Cohen’s *d*, Hedges’ *g*, or Pearson’s *r* values, and sample size. Any meta-analyses that used other measures of effect size (eg, odds ratios), articles that were qualitative reviews (meta-syntheses) or did not provide data for each individual study were removed. In addition, conference abstracts and letters to the editor were also excluded. After assessing for eligibility, there were 88 remaining meta-analyses (Figure 1).

### Data Analysis

R version 3.6.0 (R Core Team, 2019) was used to conduct analyses. The absolute value of the negative effect sizes was used, as the goal of this study was to determine the distribution, rather than direction, of effect sizes. Cohen’s *d* values were converted to Hedges’ *g* (Lakens, 2013; Formula 4), as these values are directly comparable to each other, and Hedges’ *g* accounts for biased estimates of effect size, especially in small sample sizes (Cumming, 2012).

Individual differences (as measured by Pearson’s *r*) and group differences (measured by Hedges’ *g*) were analyzed separately. In each case, to examine the distribution of effect sizes, a range of percentiles was calculated for all Pearson’s *r* effect sizes and all Hedges’ *g* effect sizes. The 25th, 50th, and 75th percentiles are relevant to the current study as these are the points that Cohen (1988, 1992) used as indicators of small, medium, and large effect sizes. This also follows the analyses conducted by Gignac and Szodorai (2016), Lovakov and Agadullina (2017), and Quintana (2017). That is, the 50th percentile is the median value, and the 25th and 75 percentiles are rank equidistant from the median. Percentiles were also calculated for two subsamples of the Hedges’ *g* effect sizes, where the studies were categorized as biomedical or psychosocial, based on the research topic of the meta-analysis. Histograms and density plots of the effect size distributions were also created to allow visualization of the data. To visualize any potential inflation bias, one-directional contour-enhanced funnel plots of the data were created, using the metafor R package (Viechtbauer, 2010). In these plots, the effect size is plotted against standard error with added contours (indicated by regions of red and orange) represent important levels of statistical significance (Peters, Sutton, Jones, Abrams, & Rushton, 2008). If the proportion of studies that falls within these contours (ie, .1 > *p* > .05, shaded orange, and .05 > *p* > .01, shaded red) is overly large, it suggests that research in the field may be affected by inflation bias and that many reported effect sizes are overestimates of true effect sizes, potentially due to sampling error, publication bias, and/or *p*-hacking (Ioannidis, 2008; Simmons et al., 2011). Finally, a series of a priori power analyses using the pwr R package (Champely, 2016) and the observed data were conducted to calculate the sample sizes required for future research to achieve various levels of statistical power for both individual differences and group differences (including the biomedical and psychosocial subsamples). The individual differences calculations used the pwr.r.test function, and the group differences calculations used the pwr.t.test function (two-samples type, assuming equal group sizes). All analyses used a two-tailed alpha of .05 and calculated the sample sizes required to achieve 60%, 70%, 80%, and 90% power for small, medium, and large effects (25th, 50th, and 75th percentiles of effect sizes).

## Results

A total of 4,049 effect sizes were extracted, of which 1,108 were Pearson’s *r* values, and 2,941 were Hedges’ *g* values (2,327 were categorized as being obtained from psychosocial research and 614 from biomedical research).

### Individual Differences Research

**Figure 2.**
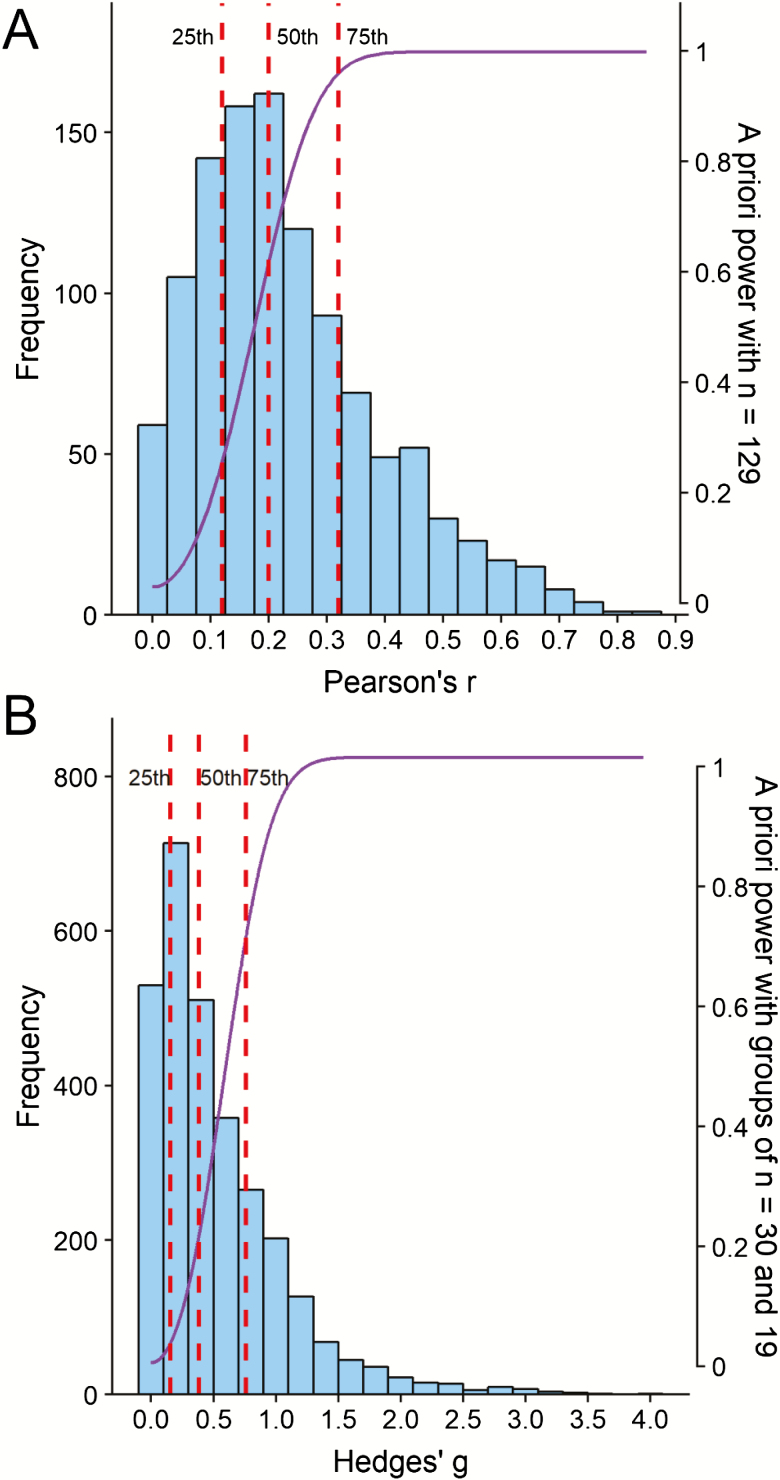
(A) The distributions of correlations (Pearson’s *r*). The dashed red lines represent the 25th, 50th, and 75th percentiles, which correspond to small (Pearson’s *r* = .12), medium (Pearson’s *r* = .20), and large (Pearson’s *r* = .32) effects. (B) The distributions of Hedges’ *g*. The dashed red lines represent the 25th, 50th, and 75th percentiles, which correspond to small (Hedges’ *g* = 0.16), medium (Hedges’ *g* = 0.38), and large (Hedges’ *g* = 0.76) effects. The purple lines in each panel represent the a priori power achieved by the median sample size of the included studies across effect sizes.

The 25th (small effect), 50th (medium effect), and 75th (large effect) percentiles corresponded to Pearson’s *r* values of .12, .20, and .32, respectively (Tables 1 and 2; Figure 2A). That is, in gerontological individual differences research, the median effect size is Pearson’s *r* = .20. Although the small effect estimate is quite consistent with Cohen’s (1988, 1992) guideline of Pearson’s *r* = .10, the estimated medium and large effects are noticeably smaller than the guidelines of .30 and .50. In comparison to Cohen’s estimates, only 29% of the observed correlations would be considered as medium effects or stronger (ie, only 29% of correlations reported Pearson’s *r* ≥ .30), and only 6.9% would be considered as strong effects (Pearson’s *r* ≥ .50).

**Table 1.** Percentiles Associated With Observed Correlations (Pearson’s r) and Group Differences (Hedges’ g)

Percentile	Pearson’s *r*	Hedges’ *g*
5	.02	0.02
10	.05	0.05
15	.08	0.08
20	.10	0.12
25	.12	0.16
30	.13	0.19
35	.15	0.23
40	.17	0.28
45	.18	0.33
50	.20	0.38
55	.22	0.44
60	.24	0.51
65	.26	0.57
70	.29	0.66
75	.32	0.76
80	.35	0.88
85	.41	1.02
90	.46	1.20
95	.56	1.59

**Table 2.** Comparison of Cohen’s Guidelines and Quantitatively Derived Estimates for Effect Sizes

	Effect size		
	Small	Medium	Large
Individual differences (Pearson’s *r*)			
Cohen (1988)	.10	.30	.50
Current study (*k* = 1108)	.12	.20	.32
Group differences (Hedges’ *g*)			
Cohen (1988)	0.20	0.50	0.80
All studies (*k* = 2,941)	0.16	0.38	0.76
Biomedical studies (*k* = 614)	0.12	0.26	0.49
Psychosocial studies (*k* = 2,327)	0.17	0.43	0.84

**Figure 3.**
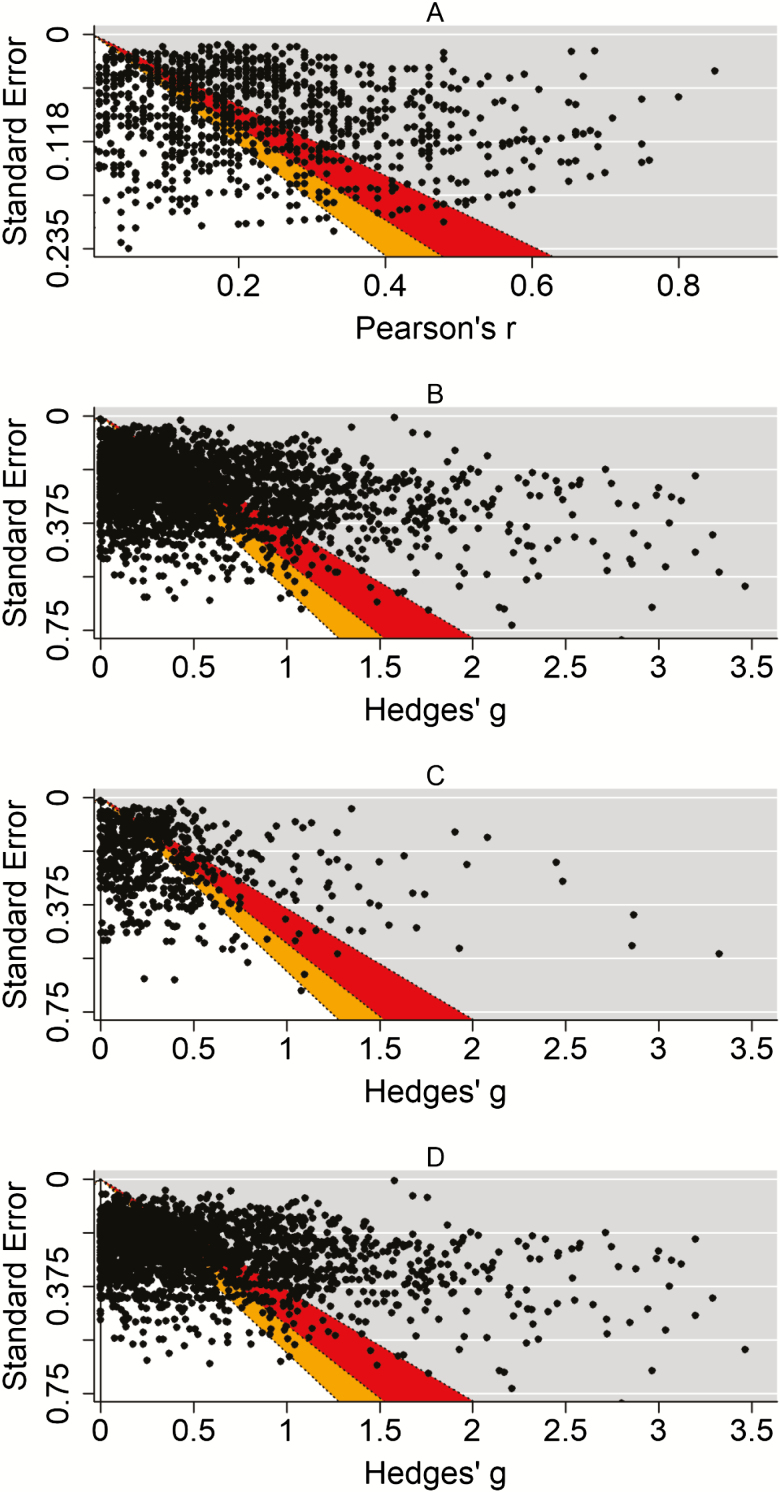
(A) One-sided contour-enhanced funnel plot for individual differences research. (B) One-sided contour-enhanced funnel plot for group differences research. (C) One-sided contour-enhanced funnel plot for group differences research in biomedical gerontology. (D) One-sided contour-enhanced funnel plot for group differences research in psychosocial gerontology.

The median individual differences sample size was 129 participants. This sample size is large enough to detect a large effect (Pearson’s *r* = .32; power = .96), but not to detect a medium (Pearson’s *r* = .20; power = .63) or small (Pearson’s *r* = .12; power = .27) effect. Only 42% (465/1,108) of the studies in the analysis were appropriately powered to detect a medium effect, although based on the contour-enhanced funnel plot (Figure 3A), there did not appear to be an overrepresentation of just-significant (*p* values between .05 and .01, represented by the red area of the figure) or marginally significant (*p* values between .10 and .05, represented by the orange area of the figure) results, suggesting that inflation bias in gerontological individual differences research is unlikely. Table 3 shows the percentages of results in each of the contoured regions of the funnel plot. Finally, Table 4 presents sample sizes required to achieve various levels of statistical power for the estimated small, medium, and large effects, using an a priori power analysis with α = .05 (two-tailed).

**Table 3.** Percentage of Results in Each of the Color Regions of the Funnel Plots

	Color region			
Funnel plot	White (*p* > .10)	Orange (.10 > *p* > .05)	Red (.05 > *p* > .01)	Gray (*p* < .01)
Individual differences (%)	28.6	6.9	13.8	50.6
Group differences (%)	49.9	6.5	11.2	32.4
Biomedical studies (%)	58.0	7.0	9.6	25.4
Psychosocial studies	47.8	6.4	11.6	34.3

**Table 4.** Sample Sizes Required to Achieve Various Levels of Statistical Power in Individual Differences Research

	Statistical power			
Effect size	60%	70%	80%	90%
Small (Pearson’s *r* = .12)	339	427	542	725
Medium (Pearson’s *r* = .20)	121	152	193	258
Large (Pearson’s *r* = .32)	47	58	74	98

*Note*. 80% statistical power is the commonly accepted level. Sample sizes were calculated using a significance criterion of α = .05 (two-tailed).

### Group Differences Research

**Figure 4.**
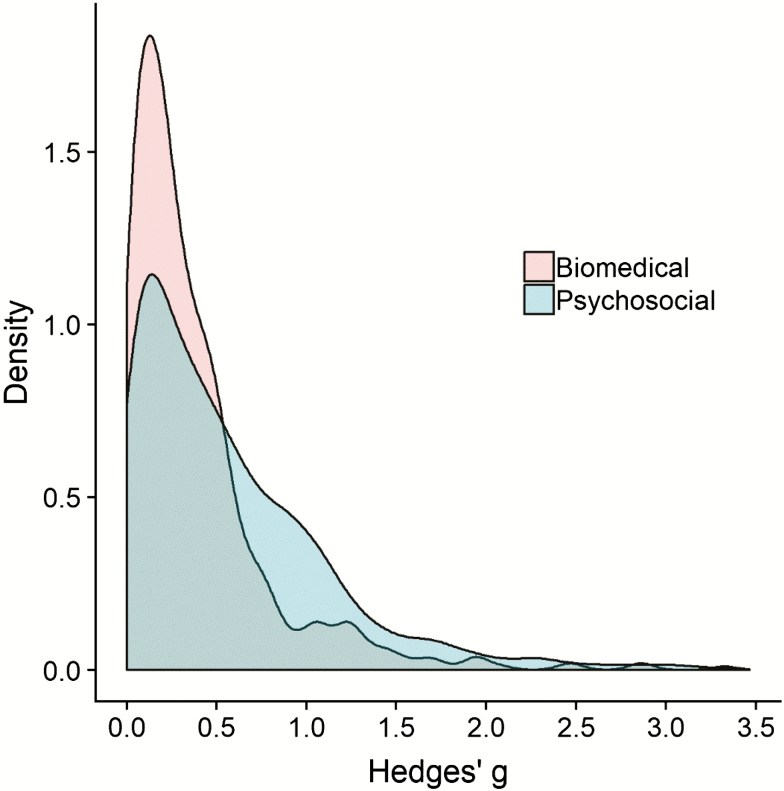
Density plots illustrating the distribution of Hedges’ *g*, based on study categorization as biomedical (pink) or psychosocial (turquoise). The distributions display the larger average effect size of the psychosocial studies.

In the group differences sample, the 25th, 50th, and 75th percentiles corresponded to Hedges’ *g* values of 0.16, 0.38, and 0.76, respectively (Tables 1 and 2; Figure 2B), which is smaller than Cohen’s (1988, 1992) guidelines of 0.20, 0.50, and 0.80. Indeed, in comparison to Cohen’s recommendation, 40.4% of the observed effect sizes would be considered as medium or stronger, and only 23.5% would be considered as large. In addition, the biomedical (estimates of 0.12, 0.26, and 0.49) and psychosocial (0.17, 0.43, and 0.84) subsamples differed greatly, and also considerably deviated from Cohen’s guidelines. Figure 4 shows a far greater concentration of small effect sizes for the biomedical (skewness = 3.79, kurtosis = 23.1) results than the psychosocial (skewness = 1.86, kurtosis = 5.22) results, which are far more dispersed, albeit still rather positively skewed.

The median sample size for case and control groups was 30 and 19 participants, respectively. This sample size is not large enough to adequately detect a large (*g* = 0.76; power = .72), medium (*g* = 0.38; power = .25), or small (*g* = 0.16; power = .08) effect (calculated with the pwr.t2n.test function of the pwr R package, which conducts power calculations for groups of unequal sizes). Furthermore, only 8% (236/2,941) of the studies in the analysis were appropriately powered to detect a medium effect. The contour-enhanced funnel plots do not show an overrepresentation of effect sizes in the significance contours, for group differences research overall (Figure 3B), nor for the biomedical (Figure 3C) or psychosocial (Figure 3D) subsamples, implying a low likelihood of inflation bias (see also Table 3). Table 5 presents sample sizes required to achieve various levels of statistical power for the estimated small, medium, and large effects, using an a priori power analysis with α = .05 (two-tailed), for group differences in gerontology overall, as well as for the biomedical and psychosocial subsamples.

**Table 5.** Sample Sizes per Group Required to Achieve Various Levels of Statistical Power in Group Differences Research

	Statistical power			
Effect size	60%	70%	80%	90%
All studies (*k* = 2,941)				
Small (Hedges’ *g* = 0.16)	402	506	643	860
Medium (Hedges’ *g* = 0.38)	67	85	107	143
Large (Hedges’ *g* = 0.76)	18	22	28	37
Biomedical studies (*k* = 614)				
Small (Hedges’ *g* = 0.12)	680	856	1,089	1,457
Medium (Hedges’ *g* = 0.26)	151	189	241	322
Large (Hedges’ *g* = 0.49)	42	53	67	89
Psychosocial studies (*k* = 2,327)				
Small (Hedges’ *g* = 0.17)	336	423	538	720
Medium (Hedges’ *g* = 0.43)	53	67	85	113
Large (Hedges’ *g* = 0.84)	15	18	23	31

*Note*. Values presented in the table represent required sample size per group to achieve various levels of statistical power. 80% statistical power is the commonly accepted level. Sample sizes were calculated using a significance criterion of α = .05 (two-tailed).

## Discussion

This study aimed to investigate the distributions of effect sizes and observed statistical power in gerontological research. Cohen (1988) proposed guidelines of effect sizes for small, medium, and large effects for both individual differences (Pearson’s *r* = .10, .30, and .50, respectively) and group differences (Cohen’s *d* or Hedges’ *g* = 0.20, 0.50, and 0.80) research but also stated that these should ultimately only be used when no specific information is available regarding the likelihood of various effect sizes. The results of this study suggest that Cohen’s (1988, 1992) guidelines may overestimate average effect sizes in gerontology, which can result in sample size calculations and interpretations of observed effect sizes that are not necessarily appropriate for the field.

This study observed effect sizes of Pearson’s *r* = .12, .20, and .32 (for individual differences research) and Hedges’ *g* = 0.16, 0.38, and 0.76 (for group differences research). These values are very consistent with those reported by Gignac and Szodorai (2016), who reported effect sizes of Pearson’s *r* = .11, .19, and .29 in individual differences research in psychology but are slightly lower than those reported by Lovakov and Agadullina (2017; Pearson’s *r* = .12, .25, and .42 in social psychology). In addition, the distribution of group difference effect sizes is very similar to those reported by Lovakov and Agadullina (2017; Hedges’ *g* = 0.15, 0.38, and 0.69), although were lower than Quintana’s (2017) estimates obtained in case–control studies of heart rate variability (Cohen’s *d* = 0.26, 0.51, and 0.88), possibly due to the wide range of research topics included in this study. Nonetheless, the estimates obtained are noticeably lower than Cohen’s (1988, 1992) guidelines. As such, it is recommended that effect sizes of Pearson’s *r* = .10, .20, and .30 and Cohen’s *d* or Hedges’ *g* = 0.15, 0.40, and 0.75 should be used as thresholds to interpret small, medium, and large effects in gerontology, respectively. These values have been rounded to the nearest 0.05 from the calculated percentiles (Table 1) for ease of use. It is likely that the observed estimates in this study vary from Cohen’s guidelines and previous research in other fields (eg, Gignac & Szodorai, 2016; Lovakov & Agadullina, 2017; Quintana, 2017) for a couple of major reasons. First, it is possible that experimental methods used in gerontology may differ from other fields of research, such as how measurements of effects of interest are conducted, and potential between-participants variability with regard to outcomes of experimental manipulations and/or naturalistic observations in a representative sample of aging adults. Second, a wide range of studies from many subfields of gerontology were included in the analyses, and it is likely that there is variability between these subfields in terms of study design (eg, cross-sectional vs. longitudinal design), study sample characteristics (eg, age, typically vs. atypically aging), and true effect size (average value and homogeneity/heterogeneity). Indeed, Figure 4 shows considerable variation between biomedical and psychosocial gerontology research, and it stands to reason that further subfield analyses would also display differences in effect size distributions.

In addition, it was found that the median sample size in individual differences research (*n* = 129) only has power of .63 to observe a medium effect size (Pearson’s *r* = .20), and only .25 power in group differences research (*n* = 30 and 19 in each group, Hedges’ *g* = 0.38). These findings are both far lower than the recommended minimum level of .80 (Cohen, 1998, 1992) and show that gerontological researchers should increase sample sizes in their studies to ensure adequate and accurate levels of statistical power. Although this is not a problem exclusive to gerontology (eg, Button et al., 2013; Dumas-Mallet, Button, Boraud, Gonon, & Munafò, 2017; Quintana, 2017; Szucs & Ioannidis, 2017), it should be a major concern and priority to those in the field (Isaacowitz, 2018; Pruchno et al., 2015) as low power weakens the strength of evidence of a research finding (Brydges & Bielak, 2019) and the probability that the finding will be successfully replicated (Maxwell, 2004). Tables 4 and 5 provide estimates for gerontological researchers to use while planning a study in the field. For example, if a researcher is conducting an individual differences study and is aiming for statistical power of .80 when expecting a medium effect size (now Pearson’s *r* = .20, rather than .30), he/she should test 193 participants—far more than the current median sample size of 129.

It should be noted, however, that there are some limitations to this study. First, the study was conducted by extracting effect sizes from published meta-analyses. Although this is an efficient method of data collection for a study of this type, it is likely that a number of effects that were not included in a meta-analysis were missed, and it is possible that some effects are included more than once, due to the large number of meta-analyses. However, the overall final sample size of 4,049 effect sizes from the 10 top-ranked gerontology journals is likely to be representative of the field as a whole. Relatedly, the results of meta-analyses are often inflated due to publication bias (Bakker, van Dijk, & Wicherts, 2012), which could imply that the results reported in the current study are overestimates, and therefore Cohen’s (1988, 1992) estimates are potentially less appropriate for gerontology research. The contour-enhanced funnel plots (Figure 4) did not show any overrepresentation of marginally significant or just-significant effects in any case. However, it should be acknowledged that many tests for publication bias, including trim-and-fill (Duval & Tweedie, 2000), *p*-curve (Simonsohn, Nelson, & Simmons, 2014), and *p*-uniform (van Assen, van Aert, & Wicherts, 2015) are inaccurate when true effect sizes are heterogeneous (van Aert, Wicherts, & van Assen, 2016; Renkewitz & Keiner, 2018), as is almost certainly the case in this study due to the wide range of meta-analyses included.

In addition, gerontology is a broad field, and there is doubtless variation of effect size distributions within the field, due to factors such as specific research area, specific measures used, and populations of interest (Cohen, 1962; Schäfer & Schwarz, 2019). That being said, it could be argued that the reported values, however general, are more appropriate for gerontological research than the guidelines proposed by Cohen (1988, 1992) because they are based on published research in the field, rather than general estimates across the behavioral sciences. In addition, the splitting of the group differences effects into biomedical and psychosocial categories based on the topic of the meta-analysis was an attempt to make these distributions more specific, but this categorization is open to biases. As such, researchers should interpret these results with a degree of caution and could consider using the overall group differences values for their power calculations and/or effect size interpretations rather than the more specific values. Researchers can also access the data and code to re-categorize the data as they see fit or to create effect size distributions of more specific research areas.

In summary, Cohen’s (1988, 1992) guidelines appear to overestimate effect sizes when applied to gerontological research. Researchers in the field can benefit from using these empirically derived estimates of Pearson’s *r* = .12, .20, and .32, and Cohen’s *d* or Hedges’ *g* = 0.16, 0.38, and 0.76 to adequately and accurately power their studies when calculating sample size before data collection. These estimates can also help researchers accurately interpret observed effect sizes relative to others in the field. By applying these observed values to their studies, gerontological researchers are more likely to report results that are replicable, and therefore, produce robust science.
